# Regulatory aspects of a nanomaterial for imaging therapeutic cells

**DOI:** 10.1007/s13346-023-01359-y

**Published:** 2023-05-20

**Authors:** Margriet van der Zee, Claudette de Vries, Marc Masa, Marta Morales, Marta Rayo, Ingrid Hegger

**Affiliations:** 1 National Institute of Public Health and the Environment (RIVM), Centre for Health Protection, Bilthoven, the Netherlands; 2 Asphalion S.L, Barcelona, Spain; 3 Leitat, Barcelona, Spain

**Keywords:** Nanomedicine, Regulatory pathway, nTRACK, Imaging, Tracking, Therapeutic cells

## Abstract

The ability to track therapeutic cells upon administration to the patient is of interest to both regulators and developers of cell therapy. The European Commission Horizon2020 project nTRACK from 2017-2022 aimed to develop a multi-modal nano-imaging agent to track therapeutic cells during development of a cell therapy. As part of this project, we investigated the regulatory pathway involved for such a product if marketed as a stand-alone product. An important regulatory hurdle appeared to be the appropriate regulatory classification of the nTRACK nano-imaging agent, as neither the definition for medicinal product nor the definition for medical device appeared to be a good fit for the purpose of the product and we were confronted with diverging views of competent authorities on the classification. As a consequence, the information requirements to fulfill before conducting a First in Human trial are not evident and can only be decided upon by closely collaborating and communicating with the relevant authorities throughout the development of the product. Moreover, standard test methods for demonstrating the quality and safety of a medicinal product or medical device are not always suitable for nanomaterials such as the nTRACK nano-imaging agent. Regulatory agility is therefore a great need to prevent delay of promising medical innovations, although regulatory guidance on these products will likely improve with more experience. In this article, we outline the lessons learnt related to the regulatory process of the nTRACK nano-imaging agent for tracking therapeutic cells and offer recommendations to both regulators and developers of similar products.

**Graphical Abstract:**

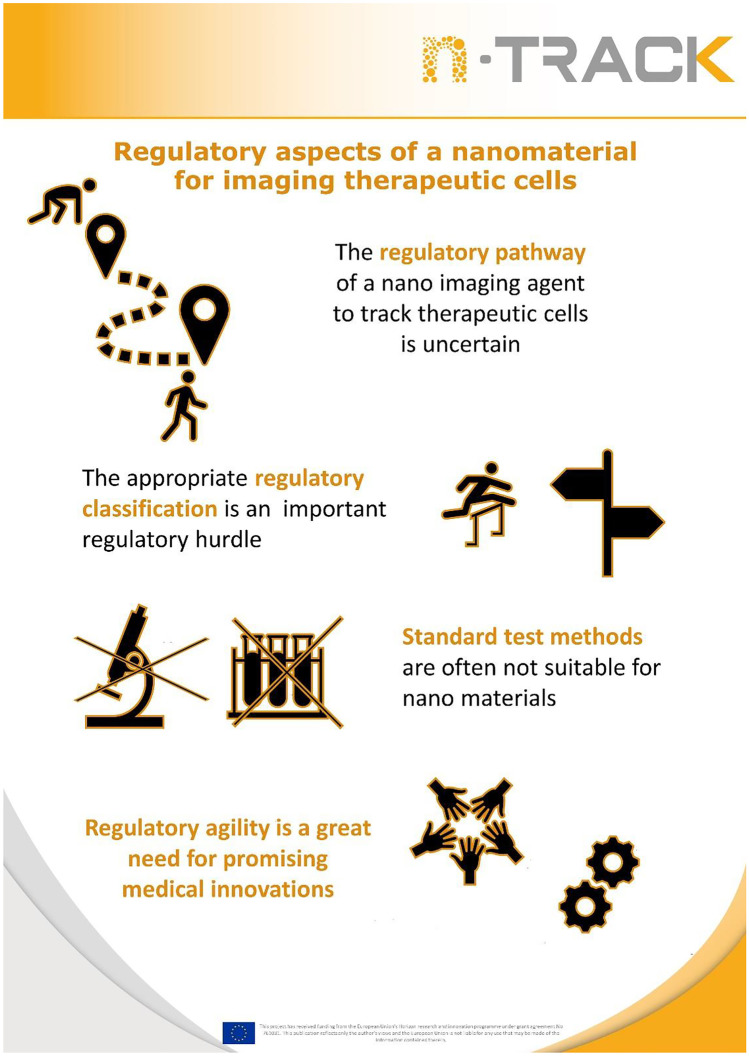

## Introduction

Many cell therapies are on the rise as a promising solution for unmet medical needs. The unknown fate of the therapeutic cells upon administration to the patient is a bottleneck and one of the decisive factors for the low number of cell therapeutic products (CTPs) that have successfully obtained marketing authorisation. The clinical development, use and marketing authorisation of CTPs would certainly benefit from more information on the distribution, viability, function and excretion of the therapeutic cells [1]. Both cell therapy developers and regulatory agencies see great merit in applying cell tracking technology [1–3].

**Fig. 1:**
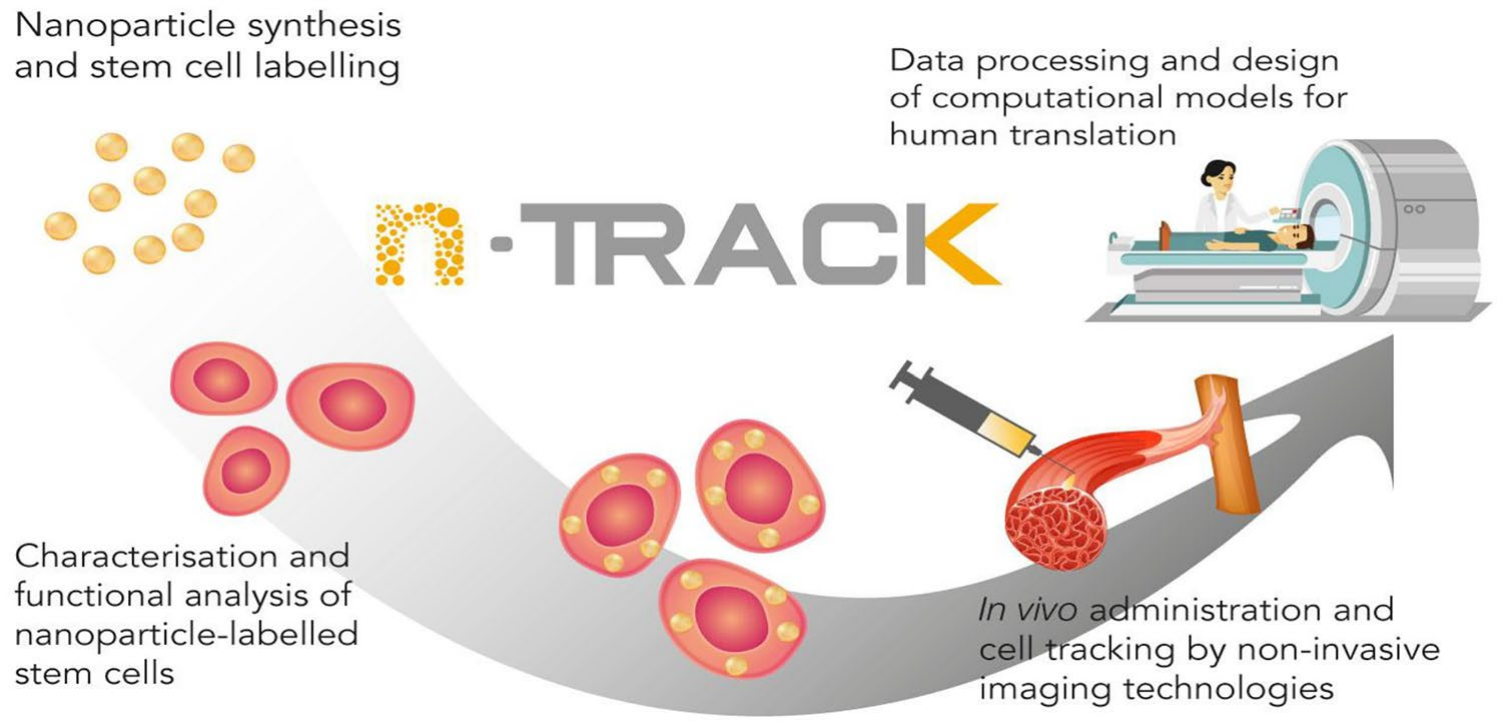
Overview of the EC Horizon2020 nTRACK project

In 2017, a European Commission (EC) Horizon 2020 funded project, nTRACK, started with the aim to develop a safe, scalable and highly sensitive nano-imaging agent to track therapeutic cells on muscle regeneration (Fig. 1). To reach this goal, the nTRACK project focussed on the development of gold nanoparticles (GNPs), because of the favourable features and scientific experience already gained with the development and clinical use of GNPs [4]. The nTRACK nano-imaging agent (hereinafter referred to as nTRACK nanoparticles) consists of glucose-coated gold nanoparticles with a primary size of 45–55 nm. The nTRACK nanoparticles are synthesised following the Enüstun and Turkevic method [5] and subsequently conjugated with PEG7 and glucose to facilitate its uptake by cells. The primary size of 45–55 nm is selected because it is shown in several studies that nanoparticles with a size of 50 nm are internalised more efficiently in cells and has a higher cellular uptake rate, than other sizes nanoparticles [6–9]. The principal intended action is that the nanoparticles are taken up by therapeutic cells, which are then administered to the patient. The nanoparticles in the cell can then be imaged by a computed tomography (CT) scan and as such provide information on the distribution of the therapeutic cells throughout the body after administration, directly and/or after a specified time period [10, 11]. The nTRACK nanoparticles are designed as a stand-alone product to be used to label any cell therapeutic product. As culture conditions and cell therapeutic products may vary, the labelling conditions must be optimised for each cell therapeutic product.

Already at the start of the nTRACK project, the consortium initiated the regulatory quality and safety evaluation activities for the nTRACK nanoparticles required for enabling a First-in-Human (FiH) trial. Due to the use of nanotechnology in combination with the challenges inherent to CTPs, we expected that the regulatory roadmap for the nTRACK nanoparticles would not be straightforward [12–15].

It is clear that the nTRACK nanoparticles in combination with CTPs are considered an Advanced Therapy Medicinal Product (ATMP) in accordance with article 2 of Regulation (EC) 1394/2007 on advanced therapy medicinal products. However, the nTRACK nanoparticles are designed as a stand-alone product to be used to track any cell therapeutic product. The question arose whether the nTRACK nanoparticles should be considered either a medical device, a medicinal product, or an excipient in the EU regulatory system. In the EU regulations, scientific data requirements for commercialization and regulatory authorities involved may significantly differ for these product categories. Therefore, one of the project’s first priorities was to determine the appropriate classification of the nTRACK nanoparticles and secondly which quality control and preclinical data requirements were necessary.

This paper summarises the main lessons learnt related to the regulatory process during the nTRACK project with the nTRACK nanoparticles as its main subject of interest. Although the focus of this paper is specifically on the nTRACK nanoparticles for tracking CTPs, much of the content is applicable to comparable tracking products as well. Therefore, findings of the project are generalised to provide recommendations for developers of cell therapy, imaging agents and regulators.

## Methods and information sources used

### Consortium partners

The nTRACK project consortium consisted of representatives of 12 institutes and companies from six countries, with a wide variety of backgrounds including nanotechnology, cell therapy, imaging, machine-learning, toxicology and regulatory affairs. At the start of the project, we consulted the consortium partners on their needs and existing know-how regarding the regulatory process applicable to nanomaterials for cell imaging using the consortium meetings, interviews and questionnaires.

### Regulatory documents and information

We reviewed a number of regulatory documents, guidelines and reflection papers for their relevance to the nTRACK nanoparticles [2, 12–29]. In addition, we looked at the regulatory product classification of other products with a similar composition and/or purpose that are on the market or have been developed previously.

### EMA-ITF

In October 2018, we held a meeting with the Innovative Task Force of the European Medicines Agency (EMA-ITF) to obtain multidisciplinary informal guidance on the development of the nTRACK nanoparticles [30]. The EMA-ITF provides a forum for early dialogue with applicants on innovative aspects in medicines development. We asked questions on the regulatory classification of the nTRACK nanoparticles and on the quality, efficacy and safety information required for a marketing authorisation of the nTRACK nanoparticles.

### National competent authorities

In the European regulatory system, clinical trials are under the remit of the National Competent Authorities (NCAs). In February 2021, we arranged a combined meeting with two NCAs with responsibilities on medicinal products, including Advanced Therapy Medicinal Products (ATMPs), and medical devices. The goal of this meeting was to seek scientific advice on the regulatory classification of the nTRACK nanoparticles and on the quality and preclinical studies required for a FiH trial with therapeutic cells combined with this agent.

### External experts

An online international workshop was held on March 23–24, 2021, which included 27 representatives from various professional backgrounds (see Table 1), to discuss the regulatory aspects encountered during the development of imaging agents for cell tracking. In preparation for the workshop, we had personal communications with representatives of an ethics committee, an imaging therapy company and a national medicines agency.

**Table 1:** Representation of the professional background and country of residence of the 27 attendees of the workshop on regulatory issues of a nano-imaging agent for tracking therapeutic cells, organised by the nTRACK project consortium

**#representatives**			
**By professional background**		**By country of residence**	
**Academic and Technical Institutes**	5	**Belgium**	1
**Consultancies**	5	**Germany**	1
**Hospital**	1	**The Netherlands**	11
**Industry**	5	**Portugal**	1
**Regulatory bodies**	11	**Spain**	8
		**UK**	2
		**USA**	3

The workshop included break-out sessions with the following topics of discussion: (1) needs and possibilities of tracking therapeutic cells; (2) regulatory classification of imaging agents; (3) quality control and preclinical data requirements; and (4) timing of regulatory steps in the research and development process.

In the next section, we will discuss these four topics in detail, using the input obtained from all information sources listed above. The views expressed in this paper should not be interpreted as binding regulatory guidance but as a preliminary set of scientific and pragmatic considerations. It should also be noted that opinions from authorities in different EU member states or from the EMA or the EU Commission may be different from the opinions described.

## Results

### Needs and possibilities for tracking therapeutic cells

#### Needs

Tracking cells after administration should provide information on the distribution of the cells in the body and how long they survive and stay in the body.

From a developers’ perspective, it is important to determine why the imaging agent is needed in the first place, for example, to optimise the dosing regimen of the cells. Furthermore, tracking cells throughout the clinical application, i.e., not just in the development phase of the therapy but also post-marketing, may aid in to determine the long-term fate of cells or to distinguish responding from non-responding patients and in further personalising the treatment. Subsequently, this determines the type of information their imaging agent needs to provide: local vs whole body, short-term vs long-term tracking, quantitative vs qualitative data, etc. Developers also need to know to what extent the imaging agent needs to be compatible with different cell types and what specific equipment is needed. At the same time, from an ethical point of view, the potential (extra) risk, for example, an anti-angiogenic effect, involved in using an imaging agent to track therapeutic cells needs to be weighed against the benefit for the patient and for cell therapy in general.

From a regulator’s perspective, any information on the fate of therapeutic cells upon administration is of added value for the marketing authorisation assessment process for a CTP; as currently, this information is often not available to regulators.

#### Possibilities

Various options for tracking therapeutic cells are available, each with their own advantages and limitations [1]. The ideal tracking method is non-invasive and uses imaging agents that are compatible with and inert to any type of (therapeutic) cells and accommodate multiple imaging techniques. In addition, the imaging agent should ideally be cleared from the body as soon as possible to prevent accumulation in organs and potential long-term adverse effects. In contrast, fast clearance of the imaging agent may not be ideal in case there is a need for long-term tracking of the cells, for example, for several weeks. As there is no fit-for-all purposes option, the method of choice depends on a number of factors such as therapeutic cell type, location of the target tissue, duration of tracking needed and availability of the required resources. The selection of the most appropriate imaging agent involves a case-by-case analysis.

#### Defining the aim at an early stage

At an early stage, researchers often tend to explore many application possibilities of their product. As the nTRACK project aimed at tracking distribution, viability and duration of the cells both during development and clinical application of cell therapies, we faced that these different application purposes may lead to a different final product (nanoparticles versus an ATMP including nanoparticles) and thus a different regulatory roadmap. This implies not only different safety and efficacy data is required, but also different regulatory authorities would be involved in obtaining marketing authorisation for the final product. Therefore, from a regulatory point of view, we recommend to define the specific aim of the nanoparticles at a very early stage of development.

The nTRACK consortium, therefore, decided to define the purpose of the nTRACK nanoparticles more precisely: ‘tracking of cell therapy with labelled stem cells to be used *during (non-clinical and clinical) development of new cell therapies*’. Initially, the imaging agent would only be used in the development phase of the cell therapy to provide input on the fate and distribution of the cells and to optimise the dosing regimen. A future option may be to develop nanoparticles for use beyond the development phase of the therapy, i.e. as an integral part of the final, marketing-authorised ATMP.

### Regulatory classification of nanoparticles

In the EU, the regulatory process and the authorities involved in approval of products with a medical purpose depend on the type of product. Medicinal products need to go through an authorisation process at the EMA or at national medicines agencies, while medical devices need to obtain a CE marking from a Notified Body. ATMPs, including CTPs, are subject to a centralised authorisation procedure at the EMA according to Regulation (EC) No 1394/2007 [24]. The CE marking for a medical device is a codification mark that implies the product has been assessed to meet high safety, health, and environmental protection requirements in accordance with the Medical Device Regulation (EU) 2017/745 [17].

To determine the regulatory classification of the nTRACK nanoparticles as a stand-alone product, we considered the EU legal definitions of medicinal product, diagnostic agent, medical device and novel excipient and assessed whether any of them were applicable to the nTRACK nanoparticles.

The nTRACK nanoparticles do not fit in the definitions of a medicinal product, as medicinal products exert their function by restoring, correcting or modifying physiological functions [16]. The nTRACK particles are used to track the therapeutical cells. Restoring the physiological function (muscle regeneration) is the intended action of the therapeutic cell, not of the nTRACK nanoparticles. Furthermore, the nTRACK nanoparticles have no diagnostic function nor are they intended to monitor a disease like a diagnostic agent [31]. The nanoparticles will be used for the purpose of monitoring the treatment of a disease and will provide valuable information which will help in the development of the treatment. Therefore, classification of the nTRACK nanoparticles as a medical device [17] may be a better fit. On the other hand as the nTRACK nanoparticles are always intended to be administrated in combination with therapeutic cells, it can be argued that there is no regulatory need to classify the nanoparticles by themselves at all, but consider them an excipient [32]. For excipients, no separate authorisation is required. Instead, depending on the dosage and route of administration of novel excipients, details of manufacture, characterisation and controls with cross-references to supporting safety data should be added to the dossier of the final medicinal product.

According to the defined purpose of the nTRACK nanoparticles, the medicinal product would be an ATMP used as an investigational medicinal product (IMP) in a clinical trial. However, the nTRACK nanoparticles will only be part of the IMP, but not of the finally marketed ATMP.

#### Authorities’ point of view

In the EU, there is no authority with the jurisdiction to assign the appropriate regulatory classification to a medical product such as the nTRACK nanoparticles. The respective authorities only determine whether a product market authorization request falls within their own legal scope or not. As a consequence, it is up to the applicants to determine a suitable classification of their product based on the definitions in the various regulatory documents and submit their application to the relevant authority. However, innovative products or novel applications of a product may not always fit well within the existing definitions of medical products, as demonstrated in the case of the nTRACK nanoparticles. In the EU, the manufacturer should select their preferred option for classification of their product and justify this to the authority in charge.

In our meetings with regulatory authorities, we found that the appropriate classification of the nTRACK nanoparticles is uncertain and that experts have different preferences for either the classification as medicinal product or medical device. We also became aware that EU Member States might have diverging views on the classification of the nTRACK nanoparticles. Examples of similar products being classified as medical devices and as diagnostic agents reflect the ongoing discussion on which visualisation purposes fall within the term ‘medical diagnosis’.

For the classification of the nTRACK nanoparticles as medical device, it became clear that the interpretation of the term ‘medical purposes’ in the definition of medical device is crucial. For example, according to the current German Medical Device Law (§ 3 MPG), the term ‘medical purposes’ currently relates to individual patient benefit and not to medical science in general Taking this into account, we anticipate that nTRACK nanoparticles probably do not qualify as a medical device in the context of current German medical device legislation. However, the interpretation of medical purposes may evolve due to the implementation of the Medical Device Regulation in the EU.

A representative from a Notified Body who attended our international workshop further argued that the Medical Device Regulation is only applicable to products that are placed on the market and used in routine clinical settings and not, as in the case of the nTRACK nanoparticles, for tools used during development (Personal communication). This implies that the particles as such will not need to be assessed and approved by a European NCA as they are neither a medical device nor a medicinal product. However, the nTRACK nanoparticles will be used as part of an IMP according to the EU legislation. Each combination of the nanoparticles and an ATMP would need to be approved as an IMP. The option to consider the nanoparticles as an excipient was brought up during the international workshop. The classification as excipient would be consistent with the opinion of the expert from the Notified Body.

In the USA, a product with a similar purpose to that of the nTRACK nanoparticles was designated a drug by the USFDA (Personal communication). Furthermore, since the product is intended to be used in combination with cells only, the jurisdiction of reviewing the product was assigned to the USFDA’s Center for Biologics Evaluation and Research (CBER). The safety and efficacy data supporting the use of the product is largely directed at the impact of the product on the therapeutic cell it is combined with. In other words, while being designated a drug, its review is handled as if it were an excipient.

It is clear that the regulatory classification of the nTRACK nanoparticles for tracking therapeutic cells during (clinical) development is a complex issue that remains to be resolved. Again, it is important to note that the final regulatory classification, process and bodies involved may be entirely different should the nanoparticles be used only during non-clinical development or during clinical use of the therapeutic cell product.

### Quality control and preclinical data requirements

#### Regulatory quality and preclinical information requirements for the nTRACK nanoparticles

Regardless of its regulatory classification, there can be no doubt that the quality, safety and reliability of the nTRACK nanoparticles need to be assessed extensively before its first use in humans. The data required may differ from one regulatory framework to another, although there are large overlaps. Below, we discuss the regulatory information requirements for medicinal products and medical devices in the EU.

#### Directives and guidelines on medicinal products, excipients and diagnostic agents

Directive 2001/83/EC relating to medicinal products for human use in the EU makes no mention of nano-specific considerations. As a consequence, should the nanoparticles be classified as a medicinal product, manufacturers would need to provide the same preclinical data as required for any conventional medicinal product, according to this directive. The required data include information on quality, safety and efficacy which is obtained by following strict technical guidelines. For example, the safety information largely needs to comply to the globally harmonised guidelines laid down by the International Council for Harmonization of Technical Requirements for Pharmaceuticals for Human Use (ICH).

Nevertheless, regulating nano-medicinal products as conventional medicinal products has given rise to concerns. Regional differences in their regulation across the globe are difficult to navigate and lead to a lack of harmonisation [33]. In addition, standardised methodologies used for conventional medicinal products are not always compatible with nano-medicinal products, resulting in uncertainties regarding their quality and safety [34–36]. Due to their particulate nature, nanomaterials tend to interact with the immune system, and it is uncertain whether the current set of preclinical safety studies for conventional medicinal products provides sufficient information for an adequate evaluation of the potential immunotoxicity of nano-medicinal products [37].

Despite the lack of specific regulations for nano-medicinal products, the EMA published a number of reflection papers on safety evaluation of nano-medicinal products [15, 20–22]. EMA’s paper on general issues for consideration regarding parenteral administration of coated nanomedicine products [15] emphasises the need for a well-defined and controlled manufacturing process supplemented with a suitable control strategy. The paper states that control and assurance of the quality of coated nanomedicine products cannot just be based upon a set of test specifications on the final product. Another paper from EMA discusses the general principles for assessing block copolymer micelle nano-medicinal products and recommends the companies to seek product-specific scientific advice on the data requirements due to the complexity of these products [22].

For novel excipients, there is no separate regulatory approval process in the EU, let alone for nano-excipients. The quality and biological safety evaluation is generally done simultaneously with the development of the drug containing the new excipient. In case the nTRACK nanoparticles are considered an excipient, this would mean that mainly its quality and safety are evaluated as part of the ATMP it is combined with. One approach may then be to execute a full-blown stand-alone safety evaluation of the nanoparticles, which may subsequently be used in technical dossiers for multiple ATMPs. However, data will always be needed on the combination of the nanoparticles with the ATMP under evaluation.

#### Directives and standards for nano-medicinal devices

The EU Medical Device Regulation does mention specific requirements for nanomaterials stating that ‘In the design and manufacture of devices, manufacturers should take special care when using nanoparticles for which there is a high or medium potential for internal exposure’. These devices are classified as class IIa, IIb or III, depending on their potential for internal exposure. If the nTRACK nanoparticles (or similar) are classified as a medical device, they would be classified as class III medical devices because of their high level of internal exposure.

The ISO standard 10993–1:2018, Annex A, provides a framework for an assessment of the biological risks of medical devices and is a part of the international harmonisation of the safe use evaluation of medical devices [18]. In the framework, a number of properties to be characterised are described and it refers to ISO/TR 13014 for details on the appropriate methodology [29]. This framework is generally applicable to devices that contain, generate or are composed of nanomaterials. Nevertheless, nano-specific considerations apply, as outlined in part 22 of the standard [27]. According to this guidance, nanoparticles would most likely be categorised as ‘nano-object medical device’, and their assessment needs to include characterisation of physicochemical properties, toxicokinetics and tissue distribution and a biological evaluation.

#### (Lack of) Standardised methodologies

It is important to note that for many of the required quality parameters, standardised methods compatible with nano-medicinal products are not yet available [35, 38]. Several methods are available for measuring particle size and size distribution, but large differences exist in terms of their level of standardisation, compatibility with materials, cost, complexity and the type of information they provide [9].

ISO 10993, part 22, describes known pitfalls in toxicity testing of nanomaterials that have been assessed by the H2020 project REFINE (www.refine-nanomed.eu). The REFINE project aims to advance the regulatory science for nanotechnology-based health products, investigated to what extent standardised methods were available to fulfil the regulatory information needs for nano-medicinal products and nano-medicinal devices [36]. Analysis indicated that standard test methods did not exist for determining surface properties, kinetic properties in biological media, ADME parameters and interaction with blood and the immune system. For endotoxin determination, methods exist but need to be adapted for nanomaterials, such as the limulus amebocyte lysate (LAL)-based method for endotoxin. The LAL assay interferes with a variety of nanomaterials, potentially resulting in false positive or negative results [39]. ISO 10993, part 22, lists a number of other methods and resources for information on pyrogenicity testing of nanomaterials [27]. For many other information needs, methods still need to be validated or developed, or are at a very early stage of development. For example, the Ames test, a commonly used assay for assessing genotoxicity of soluble medicinal products, is considered unreliable for nanomaterials because of their inability to cross bacterial membranes [40].

#### Extrapolating safety evaluation data from similar products

Developers of nanoparticles may turn to a large number of experimental safety studies with chemically similar nanomaterials (or their parent compounds) to fulfil the biological safety evaluation requirements of their product. While this seems a good approach, the safety profiles of nanomaterials depend on a number of properties other than chemical structure. The regulatory bodies handling medicinal products and medical devices are well aware of this.

EMA published several guidelines for nano-medicinal products, including intravenous liposomal products [21] and intravenous iron-based nano-colloidal products [20] developed with reference to an innovator medicinal product. An important message is that subtle differences in physicochemical properties between the applicant’s product and the innovator product may substantially modify the efficacy/safety of the product, which is not detectable by conventional bioequivalence testing alone.

Similar to nano-medicinal products, ISO 10993 part 22 states that demonstrating equivalence by extrapolating existing data from other similar nanomaterials, or from the corresponding parent compound, is not considered applicable for medical devices [27]. Next to chemical composition, the nanomaterial properties can be influenced by other factors such as size, shape and surface properties of the nanomaterial and/or the source (manufacturer) of these nanomaterials, manufacturing process and storage conditions.

There are quite a few challenges to overcome when extrapolating data from one nanomaterial to another for regulatory purposes [41]. One of them is to elucidate to what extent the various physicochemical properties change the safety profile of nanomaterials and how these properties can be compared [42]. For these reasons, reliable extrapolation of safety data from studies done with nanomaterials that are not identical to the final product is very limited and will likely not be readily accepted by the authorities.

#### Lessons learned from discussions with experts

Our two meetings with experts from regulatory authorities (EMA-ITF and National Competent Authorities) were very fruitful for learning which issues may be specifically important during development of nanoparticles for imaging regardless their final classification as a medical device or a medicinal product. In the international workshop, we further discussed the quality control and preclinical data requirements for nanoparticles for imaging with other experts. Based on all expert discussions in different settings, we obtained a clear picture of the main specific regulatory issues for nanoparticles for imaging, which we describe in next section.

First of all, it is important to be aware that the batch of the nanoparticles used in the preclinical studies needs to be representative to the clinical batch. After preclinical testing, changes in the manufacturing process of the nanoparticles could better be avoided.

Furthermore, particle size distribution and data on aggregation behaviour as part of the physical stability characteristics of the nanoparticles are very important. As aggregation of nanoparticles is a concern, the size distribution measurement method has to be able to differentiate between all types of aggregation, reversible and irreversible, during the stability period. For a clinical trial application, morphology, electronic microscopy of the nanoparticles and information on endotoxin levels and sterility will be needed as well.

Depending on the cell type, therapeutic cells may or may not be modulated by the presence of the nanoparticles, in terms of both viability and function. Therefore, for a clinical trial application, extensive quality and preclinical information needs to be provided for the therapeutic cells loaded with the nanoparticles. The ability to distinguish between viable and non-viable therapeutic cells after labelling in in vivo studies would also be of great benefit. It is important to demonstrate the stability of the cells after labelling and shipping.

Another important issue is a consistent procedure in labelling the therapeutic cells with the nanoparticles. To know whether the labelling is a consistent process, information on the uptake, excretion and concentration of the nanoparticles in the therapeutic cell has to be obtained, as well as the number of nanoparticles used, and what percentage of the cells have been successfully labelled. The procedure for labelling the therapeutic cell needs to be outlined in detail in a Standard Operating Procedure.

In addition to general quality, biodistribution and safety studies, it is important to confirm that the nanoparticles do not move between cells, to ensure that the imaging agent is following the therapeutic cells and not, as is the case of the macrophages that clear the nanoparticles from the body. To address this, an option could be to include an extra arm with just the nanoparticles in the preclinical studies with the labelled therapeutic cells and study the fate and safety of the nanoparticles by themselves compared to when it is administered as part of the therapeutic cell. To see what effect the nanoparticles may have on different cell types, it is essential to include several types of human cells in the preclinical studies.

Apart from the standard studies on ADME, it will also be relevant considering if the exposure to the nanoparticles themselves can affect the patient’s underlying disease, for example, due to a potential anti-angiogenic effect.

With regard to performance, the number of nanoparticles needed to enable imaging has to be quantified and optimised. In the case nanoparticles with a long persistence in the body, the possibility that the materials will interfere with future imaging techniques needs to be considered.

If computer models are used to enhance imaging, it will also be important to validate the underlying algorithms and to assess the risk for the patient in case of a false output of the model. In general, the individual patient benefit is a critical point in both the medicinal product and the medical device law, and this needs to be addressed in detail.

The EU requires final medicinal products, including ATMPs, to be produced in compliance with Good Manufacturing Practice (GMP) [24]. For medical devices, the accepted standard for production is compliance to ISO standard 13,485 Medical devices – Quality management systems – Requirements for regulatory purposes [28].

Although both types of quality systems, GMP and ISO 13485, aim to guarantee the quality and safety of the final product, they have a rather different approach making a comparison complicated. Regardless of the classification of the nanoparticles itself, the final product administered to patients during clinical development contains a medicinal product, i.e. the cell therapy product. Therefore, both the production of the therapeutic cells and the labelling of the cells with the nanoparticles need to occur under GMP conditions.

There is no specific regulatory guidance available yet for the particular combination of therapeutic cells loaded with nanoparticles.

### Timing of regulatory steps in the research and development process of nanoparticles: step-by-step recommendations

The right timing of starting the various steps involved in regulatory approval can save a lot of resources. Preparing a regulatory roadmap early on in the development of nanoparticles for cell tracking purposes has the advantage of being able to anticipate on the regulatory information requirements. However, other steps of the regulatory process, such as executing regulatory required safety studies, should wait until an upscaled manufacturing process has been finalised, and reproducible batches of the material that meet all critical quality criteria can be produced consistently.

**Fig. 2:**
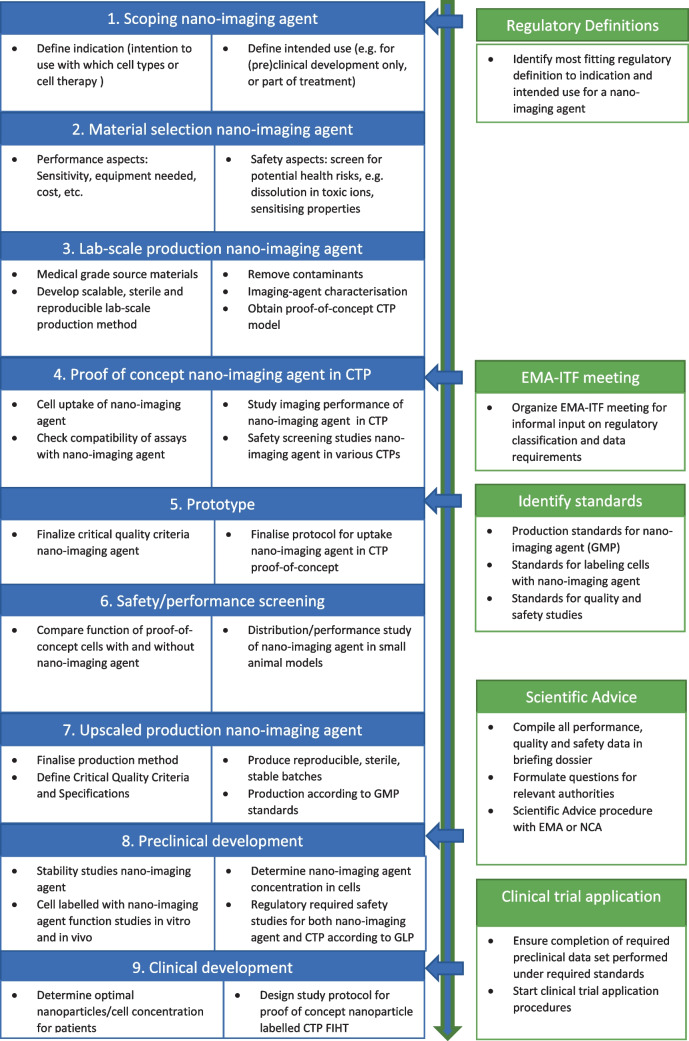
Relevant regulatory steps during development of nanoparticles for tracking therapeutic cells

The next paragraphs, summarised in Fig. 2, provide a general overview of the regulatory relevant steps in chronological order that can be executed during the development of nanoparticles for tracking therapeutic cells, up until the clinical development. The suggested order is based on experiences from the nTRACK project as well as input during the workshop and meetings held with external experts. The optimal order may change under different circumstances.

#### Regulatory classification

The first step in the regulatory process for the nTRACK nanoparticles was to define the intended primary purpose of the nanoparticles, i.e. to identify better which regulatory roadmap to follow. In general, we recommend to decide on the primary purpose of a nano-imaging agent as a first step, because this will help to identify the best fitting definition of the final product, the most appropriate regulatory process and the authorities involved in the assessment process for bringing the product to the market.

#### Meeting with experts and identifying standards

Relatively early, before the nTRACK prototype was finalised, we consulted the EMA-ITF. This gave us the opportunity to obtain the latest expert input on regulatory classification and information requirements. This information was used to identify appropriate regulatory standards for material production, quality and safety studies. However, before considering a consultation keep in mind that the best moment is when a nano-imaging agent is fully characterised, and their critical quality criteria are well-established.

Once reproducible batches of the nTRACK nanoparticles were produced, we made a selection of in vitro and in vivo assays to test safety aspects to cover different regulatory requirements independently of the regulatory framework. When selecting the assay it is important to consider which assays are really necessary, especially when animal models are used.

In the case of nTRACK nanoparticles, performance and safety studies were executed, including a thorough investigation of the potential modification of cell therapeutic function by the nanoparticles and performance studies in a small animal model. In the same study, an investigation of the distribution of the nanoparticles administered with and without the therapeutic cells was executed to identify target tissues, the latter representing a scenario where all nanoparticles are released from the therapeutic cell and into the systemic circulation. Also, cytotoxicity, reactive oxygen (ROS) induction, inflammatory potential, immunotoxicity (phagocytic activity in macrophages) and genotoxic potential of the nTRACK nanoparticles were tested.

#### Scientific advice

If nanoparticles prove to be successful in the performance and safety studies, developers can start to design a preliminary protocol for the FiH clinical trial so that the appropriate non-clinical development to obtain enough data to support the clinical trial can be defined. In addition, an upscaled production process that can manufacture a material closely resembling that of the lab-scale product needs to be developed. Especially for nanomaterials, small changes in the production process can alter the material’s properties and affect its performance. It is therefore essential that critical steps in the manufacturing process are identified, appropriate quality controls are in place and stability studies confirm the production of a material with an appropriate shelf-life before starting the FiH clinical trial.

Another essential step in the preclinical development process is that the cell uptake of the nanoparticles needs to be well-characterised and controlled. The optimal concentration of the nanoparticles in the therapeutic cell needs to be established, carefully weighing performance against the potential risk of compromising cell function with increasing concentrations of nanoparticles. The final cell loading process needs to be performed under conditions of Good Manufacturing Practices.

Both the manufacturing process and the cell loading process need to be finalised before executing the preclinical safety studies to support the FiH clinical trial. This is crucial because these studies need to be performed with the exact same product that will be used in a FiH clinical trial.

A Scientific Advice procedure with the EMA or a national competent authority will help developers to identify if the non-clinical development proposed is correct and what (additional) preclinical studies are needed, if the specifications, stability plan and manufacturing process data are sufficient for the conduction of a clinical trial and if the FIH protocol designed is appropriate.

#### Clinical trial application

The next step in the regulatory process is the clinical trial application. The clinical development can start once all required preclinical studies are completed. An investigational medicinal product dossier (IMPD) has to be compiled with all the necessary information on the manufacturing, the safety and performance studies of the nano-imaging agent alone and in combination with CTPs. The IMPD must then be submitted to the authority to start the clinical trial application procedures.

## Conclusions

Our work on the nTRACK project demonstrated that the regulatory process to authorise nanoparticles for tracking therapeutic cells during development of a cell therapy proved to be quite a challenge. Situations like this are not unique to these nanoparticles — more experience with innovative products in the medical field is often needed before regulatory processes can adapt and provide guidance.

The urgent need for this guidance is reflected by the fact that the problematic regulatory classification of the nTRACK nanoparticles and similar products potentially has a great impact on their further development and, in turn, on the development of cell therapy. Depending on the classification of nanoparticles, the regulatory authorities, procedures, information requirements and quality management systems involved differ significantly.

A certain extent of regulatory agility is therefore required to prevent delay of promising medical innovations with an unclear regulatory status reach patients and to enter the market. A helpful initiative in this regard is that the EMA has a task force dedicated on innovative medicine, which allows developers to discuss the scientific and regulatory challenges of their product in an informal setting and free of charge. Our meeting with the EMA-ITF on the nTRACK nanoparticles gave us an idea of the preclinical work to be done. At the same time, regulators were provided with some food for thought on how to handle these types of products.

Regardless of whether the imaging agent is eventually classified as a medicinal product, a diagnostic agent, an excipient or a medical device, regulatory authorities will require appropriate quality and preclinical safety information for nanoparticles used for tracking CTPs. This information needs to take into account nano-specific considerations, although the extent to which this is specified in regulatory guidance documents differs between regulatory frameworks. In general, standard methods for regulatory preclinical quality and safety studies that are compatible with nanomaterials do not always exist, but various initiatives are addressing this issue already, such as OECD’s Working Party on Manufactured Nanomaterials and EU H2020 projects REFINE-NANOMED and GRACIOUS [43–46].

Regulatory guidance on classification, quality and safety requirements for these relatively novel products is likely to improve with experience. We believe that this process can be enhanced by the active exchange of information and knowledge between different authorities and developers of such products.

## Data Availability

Not applicable.

## References

[1] Helfer BM (2021). Options for imaging cellular therapeutics in vivo: a multi-stakeholder perspective. Cytotherapy.

[2] EMA. Reflection paper on stem cell-based medicinal products. EMA/CAT/571134/2009. 2011.

[3] US-FDA. Guidance for industry: considerations for the design of early-phase clinical trials of cellular and gene therapy products, 2015. 2019.

[4] Zhang R (2023). Clinical translation of gold nanoparticles. Drug Deliv Transl Res.

[5] Enustun BV, Turkevich J (1963). Coagulation of colloidal gold. J Am Chem Soc.

[6] Lu F (2009). Size effect on cell uptake in well-suspended, uniform mesoporous silica nanoparticles. Small.

[7] Jin H (2009). Size-dependent cellular uptake and expulsion of single-walled carbon nanotubes: single particle tracking and a generic uptake model for nanoparticles. ACS Nano.

[8] Wang SH (2010). Size-dependent endocytosis of gold nanoparticles studied by three-dimensional mapping of plasmonic scattering images. J Nanobiotechnology.

[9] Clogston JD (2019). Sizing up the next generation of nanomedicines. Pharm Res.

[10] Meir R (2015). Nanomedicine for cancer immunotherapy: tracking cancer-specific T-cells in vivo with gold nanoparticles and CT imaging. ACS Nano.

[11] Betzer O (2014). Nanoparticle-based CT imaging technique for longitudinal and quantitative stem cell tracking within the brain: application in neuropsychiatric disorders. ACS Nano.

[12] Ehmann F (2013). Next-generation nanomedicines and nanosimilars: EU regulators’ initiatives relating to the development and evaluation of nanomedicines. Nanomedicine.

[13] EMA. Advanced therapy medicines: exploring solutions to foster development and expand patient access in Europe EMA/345874/2016. 2016.

[14] EMA. Reflection paper on nanotechnology-based medical products for human use. EMEA/CHMP/79769/2006. 2006.

[15] EMA. Reflection paper on surface coatings: general issues for consideration regarding parenteral administration of coated nanomedicine products. EMA/325027/2013. 2013.

[16] EU. Directive 2001/83/EC of the European Parliament and of the Council of 6 November 2001 on the Community code relating to medicinal products for human use. OJ L 311, 2001. p. 67–128.

[17] EU. Regulation (EU) 2017/745 of the European Parliament and of the Council of 5 April 2017 on medical devices, amending Directive 2001/83/EC, Regulation (EC) No 178/2002 and Regulation (EC) No 1223/2009 and repealing Council Directives 90/385/EEC and 93/42/EEC (Text with EEA relevance). OJ L 117, 5.5.2017. 2017.

[18] ISO. ISO 10993-1:2018 Biological evaluation of medical devices — part 1: evaluation and testing within a risk management process. 2018.

[19] ISO. ISO 13485: 2016 Medical Devices. 2016.

[20] EMA. Reflection paper on the data requirements for intravenous iron-based nano-colloidal products developed with reference to an innovator medicinal product. EMA/CHMP/SWP/620008/2012. 2015.

[21] EMA. Reflection paper on the data requirements for intravenous liposomal products developed with reference to an innovator liposomal product. EMA/CHMP/806058/2009/Rev. 02. 2013.

[22] EMA. Joint MHLW/EMA reflection paper on the development of block copolymer micelle medicinal products. EMA/CHMP/13099/2013. 2013.

[23] EMA. Issues identified by stakeholders: follow-up from EMA’s ATMP workshop. EMA/48099/2017. 2017.

[24] EU. Regulation (EC) No 1394/2007 of the European Parliament and of the Council of 13 November 2007 on advanced therapy medicinal products and amending Directive 2001/83/EC and Regulation (EC) No 726/2004 (Text with EEA relevance). OJ L 324 10.12.2007.

[25] ICH. ICH Guidelines. [cited 2022 13 January 2022]; Available from: https://www.ich.org/page/ich-guidelines.

[26] ISO. ISO 10993–16:2017 Biological evaluation of medical devices — part 16: toxicokinetic study design for degradation products and leachables. 2017.

[27] ISO. ISO/TR 10993–22:2017 Biological evaluation of medical devices — part 22: Guidance on nanomaterials. 2017.

[28] ISO. ISO 13485:2016 Medical devices — quality management systems — requirements for regulatory purposes. 2016.

[29] ISO. ISO/TR 13014:2012 Nanotechnologies — guidance on physico-chemical characterization of engineered nanoscale materials for toxicologic assessment. 2012.

[30] EMA. Innovation in medicines. 2021 [cited 2021 June 6]; Available from: https://www.ema.europa.eu/en/human-regulatory/research-development/innovation-medicines.

[31] EMA. Guideline on clinical evaluation of diagnostic agents (doc ref CPMP/EWP/1119/98/Rev.1 (23 July 2009) 2009.

[32] EMA. Guideline on excipients in the dossier for application for marketing authorisation of a medicinal product. EMEA/CHMP/QWP/396951/2006, in EMEA/CHMP/QWP/396951/2006, EMA, Editor. 2007.

[33] Foulkes R (2020). The regulation of nanomaterials and nanomedicines for clinical application: current and future perspectives. Biomater Sci.

[34] Halamoda-Kenzaoui B (2019). Bridging communities in the field of nanomedicine. Regul Toxicol Pharmacol.

[35] Halamoda-Kenzaoui B (2019). Mapping of the available standards against the regulatory needs for nanomedicines. Wiley Interdiscip Rev Nanomed Nanobiotechnol.

[36] Halamoda-Kenzaoui B (2021). Methodological needs in the quality and safety characterisation of nanotechnology-based health products: priorities for method development and standardisation. J Control Release.

[37] Giannakou C (2016). A comparison of immunotoxic effects of nanomedicinal products with regulatory immunotoxicity testing requirements. Int J Nanomedicine.

[38] Gioria S (2018). Are existing standard methods suitable for the evaluation of nanomedicines: some case studies. Nanomedicine (Lond).

[39] Giannakou C (2016). Immunotoxicity testing of nanomedicinal products: possible pitfalls in endotoxin determination. Curr Bionanotechnol (Discontinued).

[40] Catalan J, Stockmann-Juvala H, Norppa H (2017). A theoretical approach for a weighted assessment of the mutagenic potential of nanomaterials. Nanotoxicology.

[41] Stone V (2020). A framework for grouping and read-across of nanomaterials- supporting innovation and risk assessment. Nano Today.

[42] Park MVDZ (2018). Development of a systematic method to assess similarity between nanomaterials for human hazard evaluation purposes - lessons learnt. Nanotoxicology.

[43] Halamoda-Kenzaoui B (2022). Future perspectives for advancing regulatory science of nanotechnology-enabled health products. Drug Deliv Transl Res.

[44] REFINE-NANOMED. Regulatory science framework for nano(bio)material-based medical products and devices. 2021 [cited 2021 July 7]; Available from: http://refine-nanomed.eu/.

[45] OECD. Testing programme of manufactured nanomaterials. 2021 [cited 2021 July 7]; Available from: https://www.oecd.org/chemicalsafety/nanosafety/testing-programme-manufactured-nanomaterials.htm.

[46] GRACIOUS. Grouping nanomaterials for risk assessment. 2021 [cited 2021 July 7]; Available from: https://www.h2020gracious.eu/.

